# Research Driven by Curiosity: The Journey from Basic Molecular Biology and Virology to Studies of Human Pathogenic Coronaviruses

**DOI:** 10.1371/journal.ppat.1005023

**Published:** 2015-07-14

**Authors:** Stanley Perlman

**Affiliations:** Departments of Microbiology and Pediatrics, University of Iowa, Iowa City, Iowa, United States of America

When I began my career in biomedical research in the early 1970s, there was nothing more exciting than the findings in basic cellular macromolecular metabolism that had been recently described. The questions that were posed and the tools available for answering them seemed almost unlimited. Over time, the tools became increasingly sophisticated, making research even more exciting. This was all possible because of strong support from funding agencies, especially the National Institutes of Health and the public in general.

My research training was initially in cell biology, developmental biology, and molecular virology, but after attending medical school and receiving additional training as a pediatric infectious diseases physician, my interests became focused on immunopathology and immune evasion in viral disease. Driven partly by clinical observations, key questions for me were the following: Why did clinical disease often worsen after pathogens were cleared? Why was the host unable to efficiently titrate the immune response so that tissue damage was prevented while pathogens were cleared? I became especially interested in neurological infections after observing the devastating effects that viruses had on the developing brain. Essential to addressing these questions was identifying a useful experimental animal system. Mice infected with a murine coronavirus, mouse hepatitis virus (MHV), developed myelin (proteolipid sheath surrounding neural axons) destruction as virus was cleared, serving as a good model for immunopathological disease and, parenthetically, for studies of the human disease multiple sclerosis.

In initial studies, we showed that if suckling mice were infected with a virulent form of MHV, they succumbed to acute encephalitis. However, if the dams were immunized to MHV, infected suckling mice survived, but virus recrudesced with subsequent myelin destruction in the spinal cord. Mechanistic studies showed that escape from the cytotoxic CD8 T cell response (CTL escape) was a major component in virus recrudescence, and that this was facilitated by a poor antibody response. Even several years after its description, this remains as one of the best model infections for studies of CTL escape.

CD8 T cells are important for MHV clearance, but they did not appear to be the main factor in immunopathological disease. The second major discovery was that another type of T cell, the CD4 T cell, was the major factor in host tissue damage in MHV-infected mice, and that virus-specific CD4 T cells were responsible for much of the tissue destruction and clinical disease. These results led to questions about how the immune response is normally turned off after virus clearance to prevent tissue damage and why this was not completely successful in MHV-infected animals. The third discovery, addressing these questions, was the identification of small numbers of virus-specific regulatory CD4 T cells, which turn off harmful immune responses, especially those caused by virus-specific CD4 T cells. This observation, in turn, raised questions about why these cells were present in only low numbers in the infected host and raised the possibility that these cells represent a therapeutic option in patients with viral encephalitis or other infectious diseases with an immunopathological component.

Until the early 2000s, coronaviruses were not considered important human pathogens. However, this changed with the onset of the Severe Acute Respiratory Syndrome (SARS) in 2002–2003 and the emergence of the Middle East Respiratory Syndrome (MERS) in 2012. As in MHV-infected mice, clinical disease in both of these entities occurred as virus was cleared. Rapid progress in understanding these diseases and in developing vaccines and therapies occurred because of knowledge gained from studies of MHV and other animal coronaviruses. In one contribution, we showed that alveolar macrophages in the lungs contributed to poor CD8 T cell responses and that these responses were normalized if these cells were depleted prior to infection. Our approach was informed by previous studies showing the key role that T cells played in virus clearance in experimental MHV infections.

This journey from studies of murine coronaviruses to those of serious human pathogens illustrates the importance of research driven initially by curiosity. In the absence of public investment in research of animal and very mildly pathogenic coronaviruses, we would not have had a basis for developing prophylactic and therapeutic options in humans infected with the SARS or MERS coronaviruses. While working with pathogenic coronaviruses is critical for contributing to human health, it is not always easy. Members of my laboratory conduct all of their work under biosafety and biosecurity conditions that minimize the risk of pathogen release or inadvertent spread. Working under these conditions makes experimental manipulations more complicated and expensive, but these precautions are essential for the work. In addition to training in basic molecular virology and pathogenesis, we and others in the field have developed skills in educating governmental and healthcare authorities as well as the general public about the importance of human coronavirus research. One of the main lessons that the coronavirus community, as well as the public at large, learned from the 2002–2003 SARS outbreak was that the “fear factor” was at least as important as the actual disease. This was emphasized recently in the response to MERS research, in which some individuals focused largely on possible risks to biosafety and biosecurity posed by the studies, without considering the public health consequences of terminating ongoing studies. This response is short-sighted because it is only through basic research into these pathogens that we will be prepared for future outbreaks.

**Fig 1:**
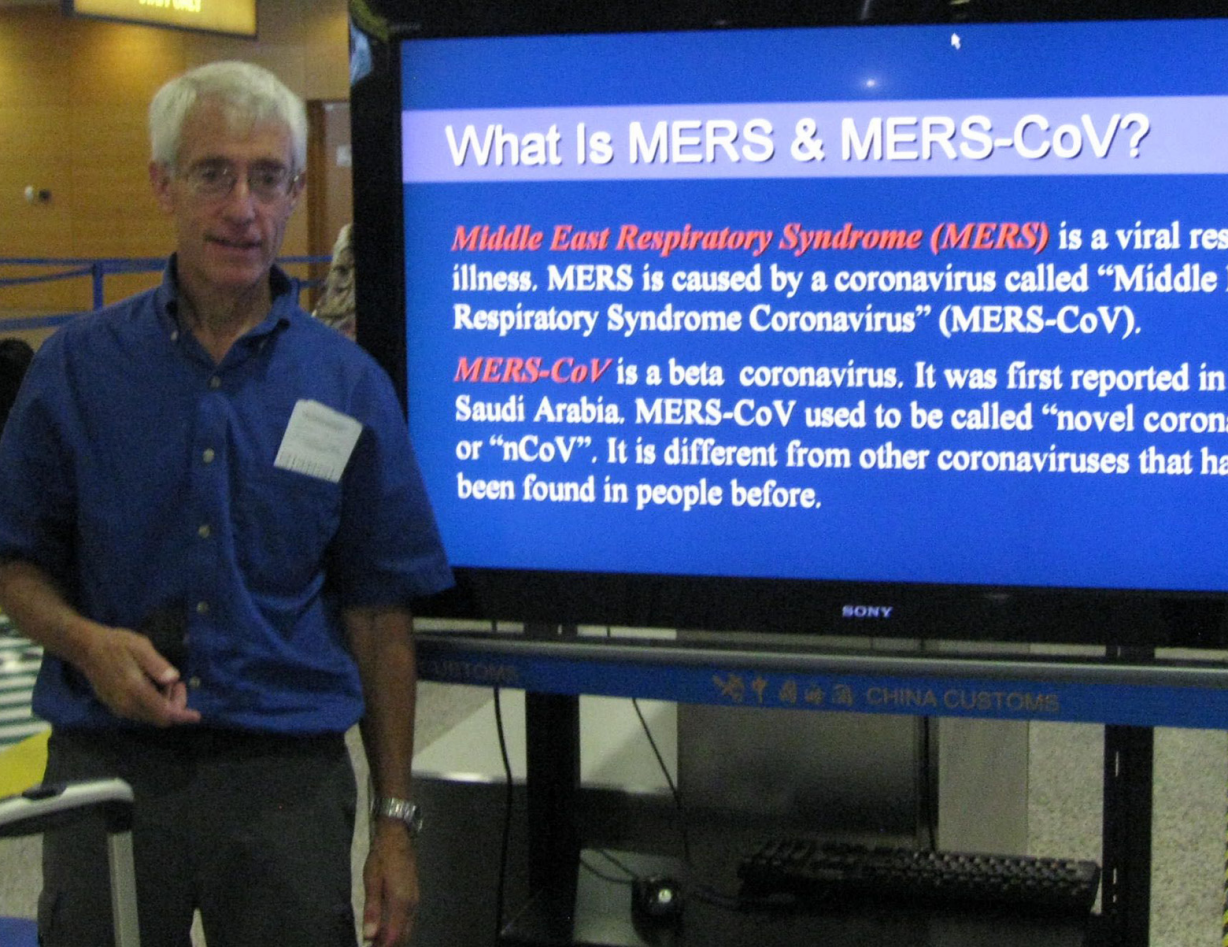
Stanley Perlman.

